# Raman spectroscopic analysis of the effect of the lichenicolous fungus *Xanthoriicola physciae* on its lichen host

**DOI:** 10.1007/s13199-016-0447-2

**Published:** 2016-10-05

**Authors:** Howell G.M. Edwards, Mark R.D. Seaward, Tom F. Preece, Susana E. Jorge-Villar, David L. Hawksworth

**Affiliations:** 1 Centre for Astrobiology & Extremophiles Research, Division of Chemical &Forensic Sciences, School of Life Sciences, University of Bradford, Bradford, BD7 1DP UK; 2 School of Archaeological Sciences, University of Bradford, Bradford, BD7 1DP UK; 3 34 Meadowbrook, Twmpath Lane, Gobowen, Oswestry, Shropshire SY10 7HD UK; 4 Area Geodinamica Interna, Facultad de Humanidades y Educacion, Universidad de Burgos, Calle Villadiego s/n, 09001 Burgos, Spain; 5 Departamento de Biología Vegetal II, Facultad de Farmacia, Universidad Complutense de Madrid, Plaza Ramón y Cajal, 28040 Madrid, Spain; 6 Department of Life Sciences, The Natural History Museum, Cromwell Road, London, SW7 5BD UK; 7 Comparative Plant and Fungal Biology, Royal Botanic Gardens, Kew, Richmond, Surrey, TW9 3DS UK

**Keywords:** Parasitism, Parietin, Pathogenicity, Protective biochemicals, Scytonemin, *Xanthoria parietina*

## Abstract

Lichenicolous (lichen-dwelling) fungi have been extensively researched taxonomically over many years, and phylogenetically in recent years, but the biology of the relationship between the invading fungus and the lichen host has received limited attention, as has the effects on the chemistry of the host, being difficult to examine in situ*.* Raman spectroscopy is an established method for the characterization of chemicals in situ, and this technique is applied to a lichenicolous fungus here for the first time. *Xanthoriicola physciae* occurs in the apothecia of *Xanthoria parietina*, producing conidia at the hymenium surface. Raman spectroscopy of apothecial sections revealed that parietin and carotenoids were destroyed in infected apothecia. Those compounds protect healthy tissues of the lichen from extreme insolation and their removal may contribute to the deterioration of the apothecia. Scytonemin was also detected, but was most probably derived from associated cyanobacteria. This work shows that Raman spectroscopy has potential for investigating changes in the chemistry of a lichen by an invading lichenicolous fungus.

## Introduction

Lichenicolous (lichen-dwelling) fungi have proved to be a major ecological group of fungi, with around 2000 species already described. Many of the genera consist only of lichenicolous species, and most are restricted to particular lichen hosts, commonly single host genera or single lichenized species. The biological interactions vary from saprobes or commensals, to gall-formers or necrotizing pathogens, but in many cases the relationships are unclear and some may even be mutualistic. Some start as pathogens, kill the host lichen, and then utilize the algal partner of the host to form an independent lichen. For more information on the variety of relationships involved see Richardson (1999), Hawksworth (2003), Lawrey and Diederich (2003), and Divakar et al. (2015).

To date, our knowledge of the interactions that take place at the biochemical or cellular level in these associations is somewhat limited (cf. Lawrey 1995, 2000; Lawrey et al. 1999; Merinero et al. 2015; Asplund et al. 2016). Microscopy has provided information on whether the fungal or the algal partner in a lichen is parasitized (de los Rios and Grube 2000) and thin-layer chromatography suggests compounds not detected in the host lichen may be produced, and perhaps originate from the invading fungus (Hawksworth et al. 1993). More sensitive methods able to examine changes in situ are needed to better explore these relationships in depth. Here we use Raman spectroscopy, which has proved particularly suitable for the molecular analysis of the protective compounds produced by lichens and cyanobacteria in stressed environments (e.g. Seaward and Edwards 1995, 1997; Russell et al. 1998; Wynn-Williams and Edwards 2000a; Edwards et al. 2004), but not previously in investigations of lichenicolous fungi.

Surface-dwelling organisms require photosynthetically active radiation in the visible region of the electromagnetic spectrum for survival, but insolation by low wavelength high energy ultraviolet radiation and exposure to high intensities of visible radiation (e.g. Solhaug and Gauslaa 2012) can be damaging unless these organisms have developed a photoprotective screening strategy (Cockell and Knowland 1999; Wynn-Williams and Edwards 2000, 2002); however, it must be noted that some authors (Robson et al. 2015; Hideg et al. 2013) have emphasised UV as a regulatory factor rather than as a stressor in such circumstances. In the case of *Xanthoria* (e.g. Gauslaa and Ustvedt 2003), photoprotection is provided by bright yellow to orange anthraquinone pigments, of which parietin predominates (Culberson et al. 1977). The amount of pigment produced varies according to the light regime of the habitat (Edwards et al. 2003a) and *Xanthoria parietina* protected under perspex cloches in an Antarctic habitat produced less parietin than similar colonies outside; specimens can vary from white through grey, yellowish-grey, yellow, orange, to orange-red with increasing light intensity, and when suddenly put in the dark they become greenish within a few days (Hawksworth and Wiltshire 2011).

Earlier Raman spectroscopic studies of *X. parietina* (Edwards et al. 2003b, 2004) identified the characteristic spectral biomarkers (19 bands) of parietin, along with associated accessory carotenoids, on several substrata in different environments. The dualistic role of important photoprotective pigments has been recognised (Cockell and Knowland 1999) and their production, along with other key lichen chemicals, in response to stressed habitats have been monitored by means of Raman spectroscopy (Edwards et al. 2003a, 2004; Wynn-Williams and Edwards 2000), its discriminatory sensitivity being used for the recognition of key biological signatures of the protective chemicals.

**Fig. 1:**
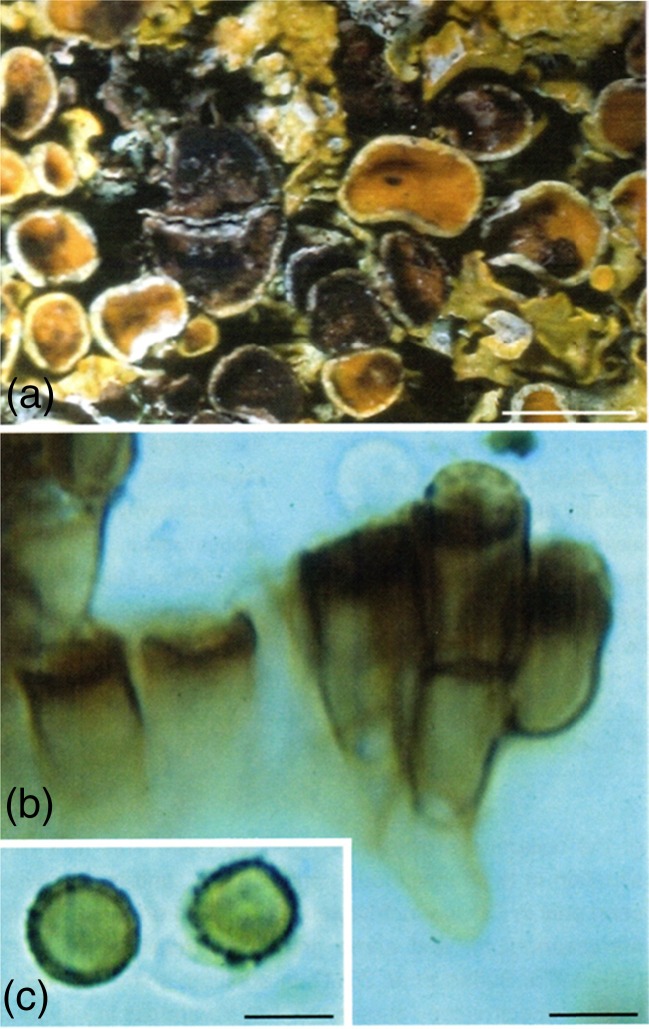
*Xanthoria parietina* thallus infected with *Xanthoriicola physciae* (K(M) IMI 164974): **a** surface view; **b** vertical section of hymenium showing conidiogenous cells; and **c** conidia. Bars: A = 5 mm, B–C = 5 μm. (Reproduced with permission from Ruibal et al. 2011)

Here we report for the first time a Raman spectroscopic analysis of *X. parietina* parasitized by the lichenicolous asexual fungus *Xanthoriicola physciae* which appears to be largely confined to Europe and is particularly frequent in the British Isles (Hawksworth and Punithalingam 1973; Hawksworth 1979; Preece 2013)*.* In the case of *Xanthoria parietina,* the invading fungus is parasitic, the hyphae growing through the host hymenium and forming conidiogenous cells just below the surface, with conidia at the surface (Fig. 1). Extensive sooty black discoloration occurs. The association is specialized, the fungus evidently being restricted to a single host species, even when growing adjacent to other *Xanthoria* species such as *X. polycarpa*. The fungus is normally found infecting groups of 5–10 apothecia, often adjacent to partially or unaffected apothecia; therefore only parts of a thallus are generally parasitized, and long-term observations on particular thalli (Preece, *unpubl*.) indicate that larger apothecia are preferentially affected and that adjacent healthy colonies can overgrow the infected areas; the new colonies can remain unaffected by the lichenicolous fungus present on the parasitized thalli beneath them.

In order to become established, the invading fungus must circumvent the protective systems of the host, both structural and chemical, as well as being capable of withstanding environmental stresses. In this connection, it is interesting to note that molecular phylogenetic studies have revealed that the closest known relatives of *Xanthoriicola physciae* included rock-inhabiting fungi from Antarctica (Ruibal et al. 2011).

In earlier studies of *Xanthoria parietina* in Antarctica (Edwards et al. 2003a, 2004), at a site at the fringe of the “ozone hole”, the protective biochemical strategy of the lichen depended on the production of significant quantities of parietin and a carotenoid accessory pigment, the relative quantities of which were monitored non-destructively over time according to incident solar radiation. Chemical biomarker signatures of the lichen fully exposed to the Antarctic environment were compared with adjacent colonies protected under Perspex cloches; it was found that the production of these pigments changed quantitatively, suggesting that both the parietin and the carotenoid components function as photoprotective chemicals in the lichen’s survival strategy. We wished to ascertain whether Raman spectroscopy could also reveal changes in chemical components of *X. parietina* when invaded by the parasitic fungus *Xanthoriicola physciae.*

## Methods

Infected and uninfected samples of *Xanthoria parietina* were collected from several sites in Shropshire and Herefordshire in the UK for investigation. Raman spectra were obtained from a Bruker IFS66/FRA 106 Fourier-transform spectrometer, operating in the near-infrared at 1064 nm using a Nd^3+^ /YAG laser, at a 4 cm^−1^ spectral resolution and with a spectral accumulation of up to 1000 scans to achieve good signal-to-noise ratios; detailed precision analyses, duly replicated, could be accomplished in c. 1 h for points on the thallus or apothecium using a spectral footprint of 100 μm. A Renishaw InVia confocal microspectrometer operating with a 785 nm laser excitation and various lens objectives were used to obtain Raman spectra from footprints of 2–25 μm. Each spectrum took *c*. 25 s to scan at a resolution of 2 cm^−1^; despite the observed increased spectral background due to fluorescence emission at 785 nm compared with that using 1064 nm excitation, the advantage of probing the infected areas of the apothecial groups at a higher spatial resolution provided some additional information, particularly for areas of the thallus between infected and unaffected apothecia. The laser illumination was deliberately kept at a minimum level to avoid sample degradation; several replicates were undertaken to verify that sample damage had not occurred.

## Results

**Fig. 2:**
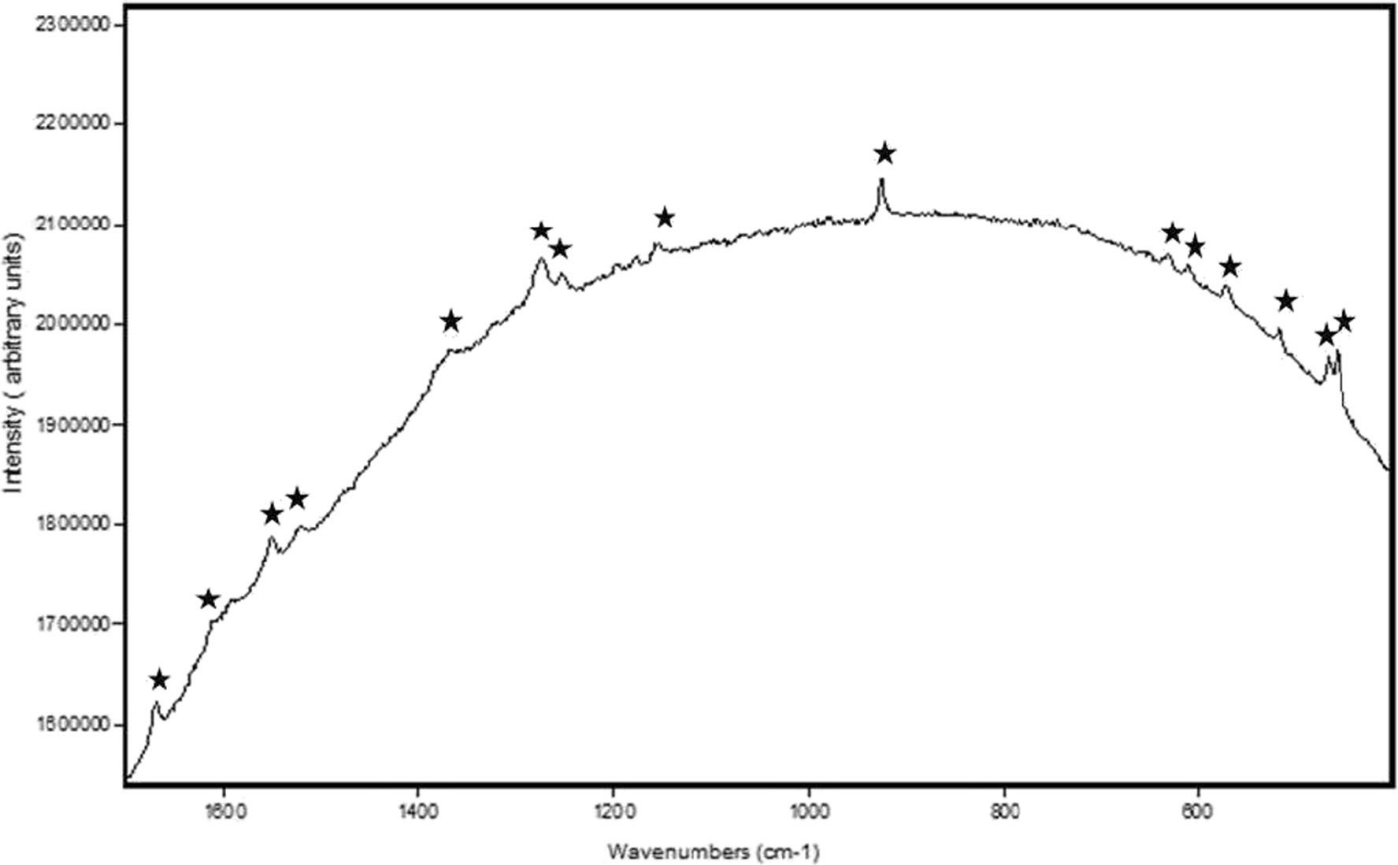
Raman spectral stackplot of healthy *Xanthoria parietina* and parietin (1064 nm excitation, 4 cm^−1^ resolution, range 200–1700 cm^−1^). *Asterisks* highlight spectral bands characteristic of parietin

**Fig. 3:**
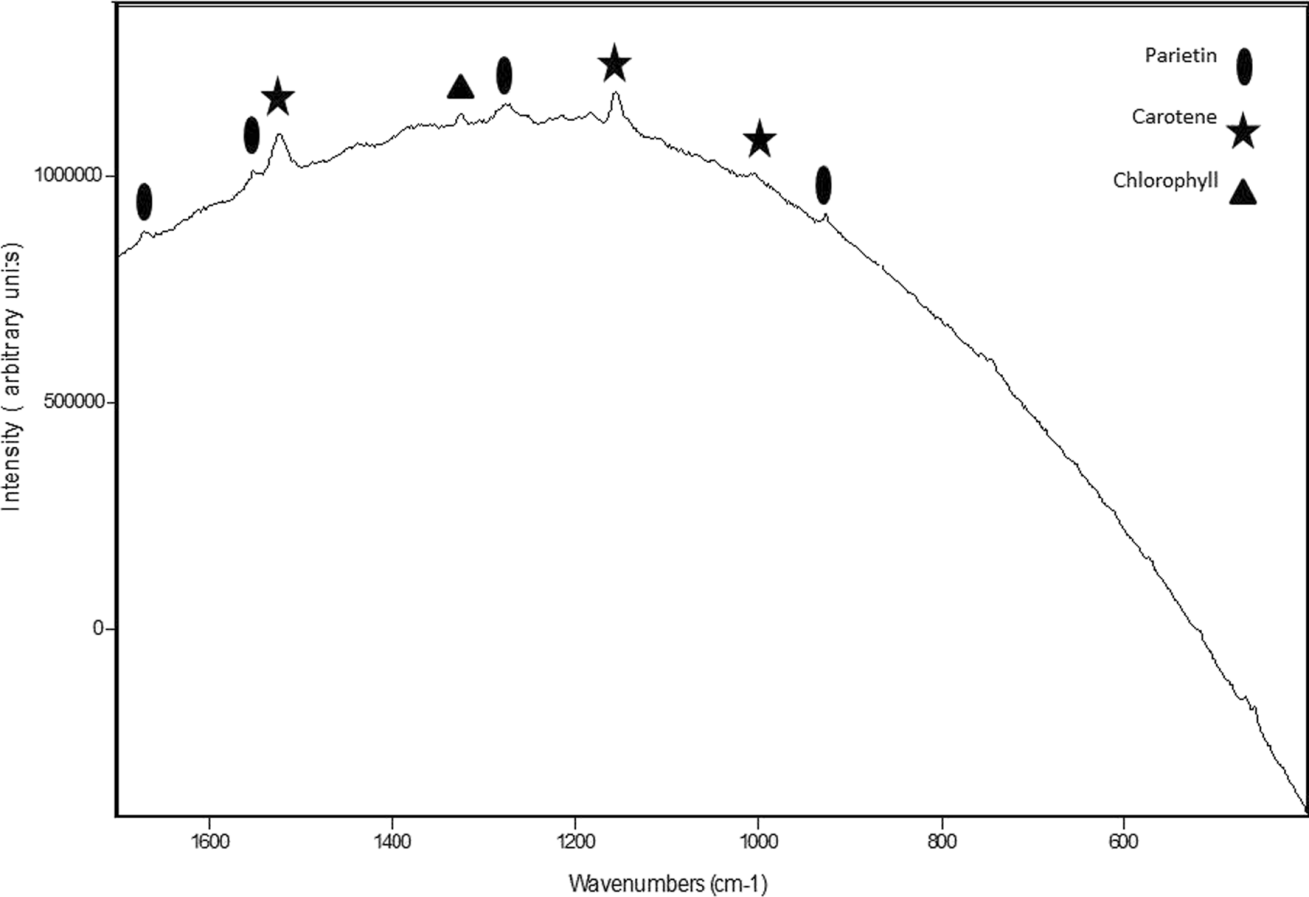
Raman spectrum (range 200–1700 cm^−1^) of *Xanthoria parietina*, with the major features of parietin, carotene, and chlorophyll indicated. The three strong features at 1527, 1154 and 1003 cm^−1^ are assignable to the carotenoid accessory pigment found with parietin in healthy tissues of the lichen

The Raman spectrum of uninfected *Xanthoria parietina* specimens (Fig. 2) is shown with the characteristic features of parietin indicated with an asterisk over the wavenumber range 200–1700 cm^−1^; this spectrum has some residual background fluorescence on which the Raman spectral bands are superimposed and demonstrate that the major chemical component in healthy *X. parietina* is the anthraquinone pigment, parietin. In these spectra, no correction has been applied for spectral background subtraction as this can often give an artificial distortion of band profiles although enhancing the band intensity and increasing spectral noise. Figure 3 shows the Raman spectrum of *X. parietina* in which four of these characteristic parietin bands have been identified. Three other features in this spectrum can be assigned to a carotenoid with bands at 1527, 1154 and 1003 cm^−1^, these being the C = C, C-C and C = CH modes, respectively, of the unsaturated carotenoid chain (Fernandes et al. 2015; Withnall et al. 2003). The band assignments are given in Table 1; from the position of the C = C stretching band it is possible to identify the carotenoid as zeaxanthin. One other weaker band near 1325 cm^−1^ can be assigned to chlorophyll. The specimens analysed for Fig. 2 clearly have much smaller concentrations of carotenoid and chlorophyll than those which generated the spectra in Fig. 3.

**Table 1:** Raman band wavenumbers and vibrational assignments of parietin pigment

Observed band wavenumber /cm^-1^	Approximate description of vibrational mode
1671	CO stretching, anthraquinone
1613	CCH aromatic ring quadrant stretching
1590	CC aromatic ring stretching
1553	CC aromatic ring stretching
1480	CC stretching coupled with aromatic phenol
	OH deformation
1370	CO phenyl stretching
1277	CC in-plane ring stretching
1255	CO aromatic ring
1198	CCC ring stretch
1180	CCC ring stretch
926	CCH out-of-plane deformation
631	CCC skeletal deformation
612	CCC skeletal deformation
570	CCO deformation
460	CCO deformation

**Fig. 4:**
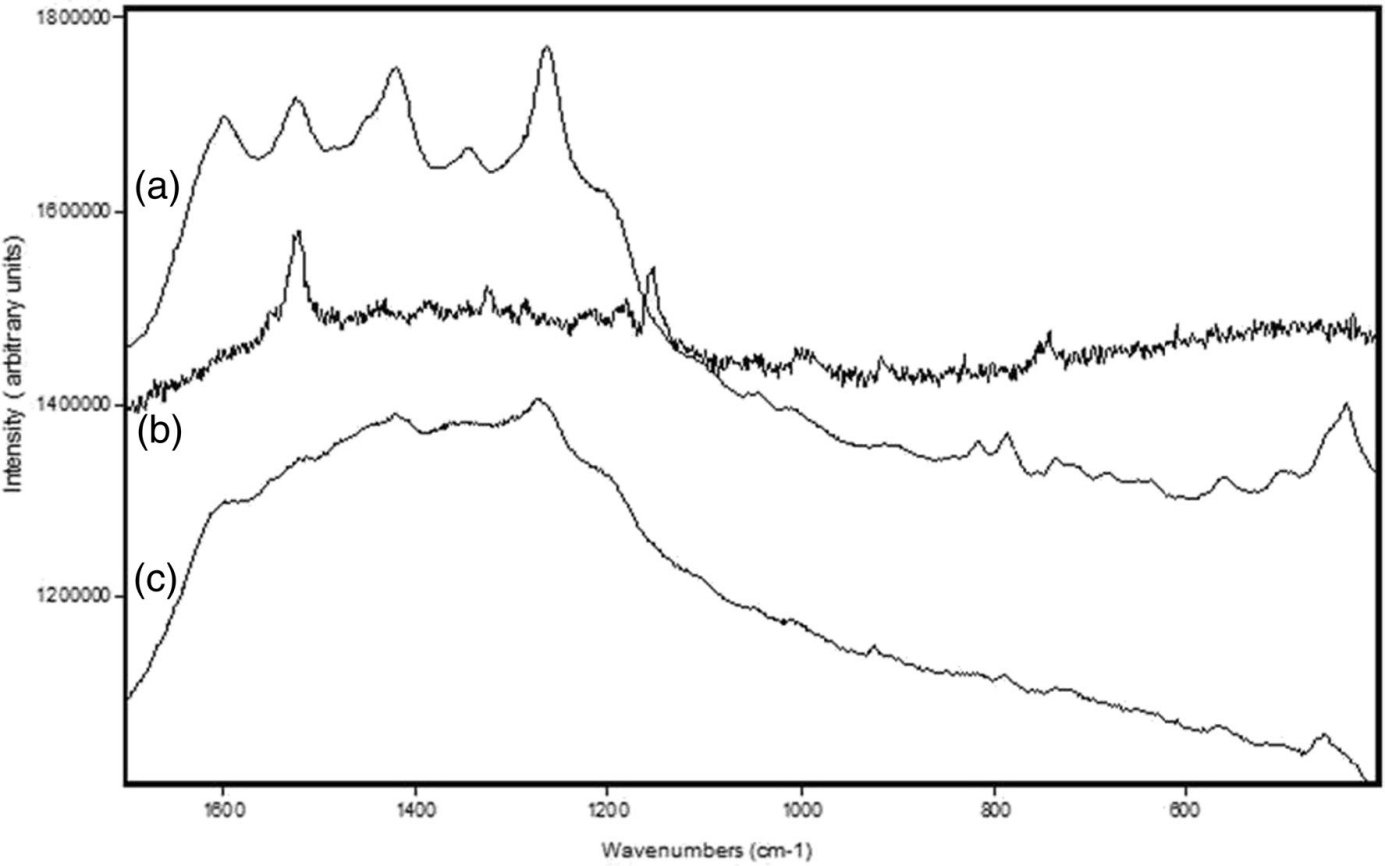
Raman spectra (785 nm excitation, range 100–1800 cm^−1^) of: **a** the blackened apothecia of infected *Xanthoria parietina*; **b** the thallial zone between healthy and infected apothecia; and **c** the brown coloured region at the centre of partially blackened infected apothecia

The Raman spectrum of *X. parietina* was also obtained using 785 nm excitation and a confocal Raman microscope, with a similar result to that obtained at 1064 nm, but with changes in relative band intensities reflecting different instrumental detector responses; however, despite the presence of a significantly increased fluorescence background emission at 785 nm, it was necessary to use confocal Raman microscopy to examine the infected specimens of *X. parietina* as this gave the spatial resolution required to distinguish the thallial zone between the infected blackened apothecia and uninfected host tissue. The Raman spectrum of the blackened apothecia resulting from *Xanthoriicola physciae* infection (Fig. 4a) is different from that observed for the unaffected zones in that the characteristic spectral signatures of parietin and the carotenoid are now absent; there are also major bands at 1598, 1522, 1421, 1343, 1264, 1206 and 433 cm^−1^ with several weaker features at 1450, 1045, 1015, 911, 816, 786, 735, 560 and 500 cm^−1^ which are characteristic of scytonemin, the radiation protective pigment found in black cyanobacterial colonies in the stressed environments of hot and cold deserts. Scytonemin is effective in the suppression of high energy ultraviolet radiation and is synthesised exclusively in the outer sheaths of cyanobacteria (e.g. Büdel et al. 1997; Dembitsky and Srebnik 2002) and is not normally therefore found associated with lichen or fungal pigments. It has been fully characterised hitherto by Raman spectroscopy (Varnali et al. 2009) and its presence noted alone and in admixture with other pigments such as carotenoids and chlorophyll (Edwards et al. 2005).

The thallial zone between the infected and healthy apothecia of *Xanthoria parietina* lacks the intense orange-yellow pigmentation and is therefore predominantly pale yellowish-grey; the Raman spectrum of this region (Fig. 4b) exhibits a different pattern from both the healthy and infected areas. However, although the spectral signatures of the parietin are absent, those of the carotenoid are still present along with several characteristic, although of weaker intensity, bands of chlorophyll at 1437, 1374, 1324, 1285, 1213 and 743 cm^−1^.

Finally, in the Raman spectrum of the brown coloured region situated at the centre of several infected apothecia (Fig. 4c), neither the parietin or carotenoid components are evident, but the spectrum is still significantly different from that of the fully blackened infected apothecia (Fig. 4a). The strong bands in the blackened apothecia due to scytonemin at 1598 and 1524 cm^−1^ are absent there, but features seem to be developing at these wavenumbers alongside the strong bands at 1421 and 1263 cm^−1^ that are already present. Other bands at 1364, 923, 736, 566 and 516 cm^−1^, although of weak intensity, are identifiable in this spectrum, but the characteristic bands of both parietin and zeaxanthin are absent.

## Discussion

The Raman spectroscopic investigation of *Xanthoria parietina* infected with the lichenicolous fungus *Xanthoriicola physciae* revealed differences in the chemistry of the system at a microscopic level. The spectra of the infected apothecia show that the parietin and carotenoid pigments have been destroyed; furthermore, the spectral signatures of scytonemin present are indicative of cyanobacterial colonisation of these areas as scytonemin is exclusively produced by cyanobacteria. This is not surprising as colonies of cyanobacteria are sometimes found on uninfected apothecia and thalli of *Xanthoria parietina*, forming minute dark brown to black spots, and are sometimes misidentified as *Xanthoriicola physciicola* in the field. In the zones bordering the infected apothecia on an otherwise healthy host lichen, the presence of the carotenoid (and chlorophyll) is noted, but here again the parietin has been destroyed. Finally, several infected apothecia, although blackened, show a brownish coloured residue in their centres which indicates that scytonemin is present in minor quantity but that another component, as yet unidentified, is also present. It is a possibility, of course, that the strength of the scytonemin signal is actually masking those of the parietin and chlorophyll, but in other extremophilic colonisations of cyanobacteria studied by Raman spectroscopy (Edwards et al. 2005; Russell et al. 1998; Wynn-Williams and Edwards 2000a) the presence of carotenoids and chlorophyll as well as bioinorganic signatures from modified geological matrices have been noted clearly.

These results provide a basis for an assessment of the strategies being adopted by the parasite and its lichen host. *Xanthoria parietina* has been shown to be capable of surviving in radiation-stressed environments where the key to its survival is attributed to the production of the protective pigment parietin in association with an accessory carotenoid. The destruction of both these pigments by *Xanthoriicola physciae*, as indicated in the results obtained here, most probably contributes to the deterioration of the apothecia of its host. The production of the protective pigment scytonemin in the destroyed apothecia of the host presumably indicates that cyanobacterial colonisation has also occurred. Overall, there is evidently a controlled invasion process in which the lichen limits the extent of damage by the parasite.

This work has provided a novel insight into a parasitic attack by an obligately lichenicolous fungus upon a lichen, which has been found to result in a loss of pigmented lichen products that appear to provide protection to the host against excessive light. More importantly, it has demonstrated for the first time the potential of Raman spectroscopy to investigate changes in the chemistry of lichens at the point of invasion by lichenicolous fungi.
